# The introduction of a targeted next generation sequencing diagnostic service for MH

**DOI:** 10.1186/1471-2253-14-S1-A14

**Published:** 2014-08-18

**Authors:** Dorota Fiszer, Sarah Hobson, Nickla Fisher, Marie-Anne Shaw, Sarah Shepherd, Rachel Robinson, Ruth Charlton, Phil Hopkins

**Affiliations:** 1 Leeds Institute of Biomedical & Clinical Sciences, School of Medicine, University of Leeds, Leeds, LS9 7TF, UK; 2 Malignant Hyperthermia Investigation Unit, St James’s University Hospital, Leeds, LS9 7TF, UK

## Background

In this paper we describe how we sought approval and are implementing a diagnostic service for malignant hyperthermia (MH) using clonal targeted next generation sequencing.

Approval required submission of a gene dossier to the UK Genetic Testing Network. This document included:

1. An overview of MH and the evidence for involvement of *RYR1* and *CACNA1S*;

2. Details of the genes

3. Current diagnostic approaches

4. Proposed sequencing strategy

5. Gene coverage with proposed strategy

6. Validation strategy

7. Genetic epidemiology of MH

8. Test characteristics (sensitivity, specificity, PPV, NPV)

9. Cost benefit of new test

10. Referral criteria

Following adoption of the dossier by the UGTN and validation of the sequencing strategy in a diagnostic facility, we are now in a position to offer testing. Testing will be offered to families where MH has been confirmed by IVCT and to new index cases. The cost of the sequencing is £530, compared to £3,500 for the IVCT. For index cases, the referring physician will be advised of the pre-test probability for their patient having MH as they may consider IVCT to be more cost-effective when the pre-test probability is low.

Diagnostic reports will be issued in accordance with the joint guideline of the UK Association of Clinical Genetic Science (ACGS) and the Dutch Society of Clinical Genetic Laboratory Specialists (VKGL). Variants will be classified using a 5 class system:

**Table 1:** 5 Class System

Class	Description	Interpretation
1	Clearly not pathogenic	MH not confirmed or excluded
2	Unlikely to be pathogenic	MH not confirmed or excluded
3	Variant of unknown significance (VUS)	MH not confirmed or excluded
4	Likely to be pathogenic	Consistent with diagnosis
5	Clearly pathogenic	Confirms diagnosis

Reports for classes 1 – 3 will advise IVCT.

Variants will be assigned to a class depending on their reported frequency in databases (dbSNP, 1000 Genomes, EVS), segregation analysis and functional analysis.

